# Trophic and proliferative effects of Shh on motor neurons in embryonic spinal cord culture from wildtype and G93A SOD1 mice

**DOI:** 10.1186/1471-2202-14-119

**Published:** 2013-10-11

**Authors:** Xiaoxing Ma, Patrick Turnbull, Randy Peterson, John Turnbull

**Affiliations:** 1 Department of Medicine, McMaster University, 1200 Main St West, Hamilton, ON L8N 3Z5, Canada; 2 Present address: Faculty of Applied Health Sciences, Brock University, 500 Glenridge Ave, St. Catharines, ON L2S 3A1, Canada; 3 Present address: Biotechnology Group, Activation Laboratories, 1336 Sandhill Drive, Ancaster, ON L9G 4 V5, Canada

**Keywords:** Sonic hedgehog, Primary cilium, Motor neuron, Amyotrophic lateral sclerosis

## Abstract

**Background:**

The developmental morphogen sonic hedgehog (Shh) may continue to play a trophic role in the support of terminally-differentiated motor neurons, of potential relevance to motor neuron disease. In addition, it may support the proliferation and differentiation of endogenous stem cells along motor neuronal lineages. As such, we have examined the trophic and proliferative effects of Shh supplementation or Shh antagonism in embryonic spinal cord cell cultures derived from wildtype or G93A SOD1 mice, a mouse model of amyotrophic lateral sclerosis.

**Results:**

Shh supported survival, and stimulated growth of motor neurons, neurite outgrowth, and neurosphere formation in primary culture derived from both G93A SOD1 and WT mice. Shh increased the percentage of ciliated motor neurons, especially in G93A SOD1 culture. Shh-treated cultures showed increased neuronal proliferation compared to controls and especially cyclopamine treated cultures, from G93A SOD1 and WT mice. Moreover, Shh enhanced cell survival and differentiation of motor neuron precursors in WT culture.

**Conclusions:**

Shh is neurotrophic to motor neurons and has mitogenic effects in WT and mSOD1 G93A culture in *vitro*.

## Background

The role of sonic hedgehog (Shh) in the patterning of embryonic motor neurons is well established [1-4], as is a role for Shh in the maintenance of stem cell populations in the adult [5,6]. The importance of Shh in terminally-differentiated neurons is less studied. Nonetheless, Shh signaling remains active in these cells, and Shh signaling may be of importance in adult neurodegenerative diseases including Parkinson’s disease [7-11], chronic diabetic neuropathy [12], and amyotrophic lateral sclerosis (ALS) [13].

With respect to ALS, we have shown [13] that Shh has trophic effects in cultured N2A cells transfected with plasmids expressing either human wildtype (WT) or G93A SOD1 (mSOD), a mutated human SOD1 responsible for some familial ALS. Canonical Shh signaling occurs through the primary cilium, and we have shown [14] that primary cilia are reduced in cell culture and in motor neurons in situ in the spinal cord of transgenic mSOD mice, possibly contributing to a reduction in Shh signaling in these cells.

The major clinical deficit in ALS results from the dysfunction and death of motor neurons, and therapeutic manipulation of the Shh pathway could be trophic to dying motor neurons and reduce this death. In principle, Shh could also act as a proliferative agent leading to the expansion of endogenous motor neuron progenitors and their subsequent differentiation into motor neurons, serving to replace dying motor neurons. Our previous experiments were undertaken in N2A cells, which are immortalized cells derived from mouse neuroblastoma, and not suited to the study of Shh-induced differentiation. As such, we have undertaken the following experiments in primary mixed cultures from spinal cord of embryonic WT or mSOD mice to determine the trophic and proliferative effects of Shh augmentation, or antagonism with cyclopamine.

We demonstrate here that Shh has trophic activity encouraging neurite outgrowth and prolonging survival of spinal motor neurons derived from WT and mSOD mice, and as well, proliferative activity increasing stem cell expansion and differentiation down motor neuronal lineages.

## Methods

### Animals

All breeding and animal experiments were approved by the McMaster University Animal Research Ethics Board and were carried out in accordance with guidelines of the National Institutes of Health and the Canadian Council on Animal Care. Six to eight week old male mSOD mice were mated with eight to ten week old female B6SJL mice purchased from Jackson Laboratories. We checked daily for a vaginal plug, and considered embryonic age as E 0.5 days when one was first seen. At E 13.5 days the dams were euthanized and embryonic spinal cords dissected. Embryo tails were genotyped for the mSOD transgene using the PCR protocol outlined on the Jackson Laboratories website.

### Primary cell culture

Primary mixed cultures enriched for motor neurons were undertaken as previously described [14,15]. Embryonic spinal cords were carefully dissected under microscopy and were processed individually. The isolated spinal cords with meninges removed were cut into small pieces and dissociated in 1% trypsin (Sigma) for 15 minutes. After trypsinization, an equal volume of trypsin inhibitor (Sigma) was added and the mixture was lightly triturated until a single cell suspension was achieved. The cell suspension was then transferred to neurobasal medium containing 1% glutamax (Invitrogen) and centrifuged at 400 g for 5 minutes without brake. The supernatant was discarded and the cell pellet resuspended in complete neurobasal medium containing 1% glutamax, 3% horse serum, 1× B-27 supplement (all from Invitrogen), 5 ng/ml ciliary neurotrophic factor (CNTF) and 5 ng/ml brain-derived neurotrophic factor (BDNF) (Leinco). 2.5 or 5 × 10^4^ cells per well were plated onto poly-D-lysine (Sigma) coated 8-well chamber slides (Labtek) and grown in a 37°C incubator in 5% CO_2_ environment. Unless otherwise specified, half of the culture volume was replaced with fresh medium every the third day.

### Shh and cyclopamine administration

At day 2 in culture (d2), either recombinant human Shh protein (Leinco) or cyclopamine (Sigma) was added to complete neurobasal medium. Cells grown in complete neurobasal medium served as controls. The recombinant Shh protein encompassed amino acids Cys24-Gly197 of the N-terminal portion with Ile-Ile substituted for Cys24, and was resuspended in distilled H_2_O without any carrier protein. Stock solutions of cyclopamine were diluted in pure dimethyl sulfoxide (DMSO). Even though the final amount of DMSO added to the cultures was minuscule, we initially carried DMSO controls. There was no effect of the DMSO, and normal controls (without Shh or cyclopamine) were subsequently carried as above.

### Motor neuron survival assay

To study the effect of Shh or cyclopamine on motor neuron survival, 2.5 × 10^4^ cells per well from 2 embryos of WT mice were plated. At d2, Shh (250 ng/ml) or cyclopamine (12.5 μM) was added to cell culture medium. The medium was replaced every 2 days containing Shh or cyclopamine until d11. At d11, the cell cultures were processed for immunofluorescent staining with the neurofilament heavy antibody (SMI32), a marker of motor neurons. The number of SMI32+ cells per well was counted under widefield deconvolution microscopy (Leica DMI 6000 B, Germany) at 20× magnification. 7–8 fields were counted and counts averaged.

### Assessment of motor neurons and motor neuron ciliation

In this experiment, 2 embryos from WT mice and 2 from mSOD mice were used. With each embryo, 5 × 10^4^ cells per well were plated onto poly-D-lysine coated 8-well chamber slides. At d2 of culture, the cells were treated with Shh (0, 250, 500 ng/ml) for 10 days. Half of the culture volume was replaced every third day. At d11, the cell medium were removed, fixed with 4% paraformaldehyde (PFA), and processed for immunofluorescent staining to determine the percentage of motor neurons and motor neuron ciliation. Adenylyl cyclase type 3 (ACIII) was used as a marker of primary cilia, while SMI32 was used to label motor neurons. Motor neuron numbers were estimated by counting SMI32+ cells, and motor neuron percentages calculated relative to DAPI stained nuclei. Motor neuron ciliation was assessed as the percentage of SMI32+ cells staining with ACIII.

### BrdU (bromodeoxyuridine) labeling for cell proliferation, cell survival, and cell differentiation

Cell proliferation was determined by BrdU (Sigma) incorporation. 5 × 10^4^ cells per well were plated onto poly-D-lysine coated 8-well chamber slides. At d2 of culture, the cells were treated with Shh (250 ng/ml for G93A SOD mice or 500 ng/ml for WT mice) or cyclopamine (5 μM for G93A SOD mice or 10 μM WT mice) for 10 days. Half of the culture volume was replaced every third day. At d11, 5 μM BrdU was added to the medium. For cell proliferation assays, cells were incubated with BrdU for 24 hours, after which the cell medium was removed, fixed with 4% PFA, and processed for immunofluorescent staining to determine the percentage or number of BrdU labeled cells. For cell survival and cell differentiation, the cells were incubated with BrdU for 72 hours and then BrdU was removed. The cells were continued in medium containing Shh (500 ng/ml) till d26. The cells were then fixed with 4% PFA prior to immunofluorescent staining for BrdU, oligodendrocyte transcription factor 2 (Olig2), homeobox gene Hb9 (Hb9), and SMI32.

### Immunofluorescent staining for primary cilia, motor neurons, and BrdU labeling cells

At the indicated time points, cells were fixed with 4% PFA at room temperature for 10 minutes, after which cells were washed twice with PBS (phosphate buffer solution). For detection of BrdU labeled cells, cells were pre-treated with deionized formamide for 2 h at 65°C, 2 N HCl for 30 min at 37°C, and 3% normal goat serum (Vector Laboratories) for 30 min at room temperature. For all other staining, cells were permeabilized with methanol for 10 minutes at −20°C. Fixed permeabilized cells were blocked with 5% goat serum (Invitrogen)/0.3% Triton X-100 (Sigma) in PBS for 1 hour at room temperature. All cells were then incubated with primary antibodies overnight at 4°C. Primary antibodies include rabbit anti-ACIII (1:1000, Santa Cruz Biotechnology), rat anti-BrdU (1:200, Serotec, Martinsried, Germany), mouse anti-nestin monoclonal antibody (1:200, Millipore), mouse anti-SMI32 (1:1000, Abcam), mouse anti-Olig2 polyclonal antibody (1:200, Millipore), rabbit anti-SMI32 (1:200, Abcam), rabbit anti-mouse Gli3 (1:200, Abcam), chicken anti-mouse glial fibrillary acidic protein (GFAP) (1:200, Millipore). The next day, cells were rinsed in PBS and then incubated with secondary antibodies for 4 h at 4°C. Secondary antibodies include Alexa Fluor 488 goat anti-rat antibody (1:500), Alexa Fluor 568 goat anti-mouse highly cross-adsorbed antibody (1:500), and Alexa Fluor 647 goat anti-chicken antibody (1:500) (all from Molecular Probes, Carlsbad, USA). Then cells were rinsed several times and mounted on slides and coverslipped with ProLong anti-fade with DAPI (Molecular Probes, Carlsbad, USA) and examined by widefield deconvolution microscopy (Leica DMI 6000 B).

### Cilial and cell counting

100 SMI32+ cells per treatment, an equal number from each embryo, were randomly selected to count ciliated motor neurons at 63× magnification. Only cells separate enough to be easily distinguished one from another were selected. For BrdU labeled proliferating cells, 8 to 11 random fields were captured during one session with constant camera settings (Hamamatsu Orca ER-A) at 20× magnification. For BrdU labeled surviving and differentiating cells, 15 random fields containing BrdU labeled cells were imaged at 63× magnification. (Higher magnification was used for the chronic studies since some of the BrdU label was diluted due to cell divisions, and was easier seen at higher power). Images were converted to TIFF files by using image analysis software (Image J). Single, double or triple labeling cells were counted by using LSM5 image browser (Carl Zeiss).

### Statistical analysis

A one-way analysis of variance (ANOVA) (Statistica, version 6.0, StatSoft, Tulsa, OK) was used to determine significant differences. When there was significant difference, Newman-Keuls significant difference test was used post-hoc. Unless otherwise noted, all data are presented as means ± standard error of the mean (SEM). Significant differences were defined as p ≤ 0.05, two tailed.

## Results

### The effect of Shh modulation on motor neurons in culture

**Figure 1:**
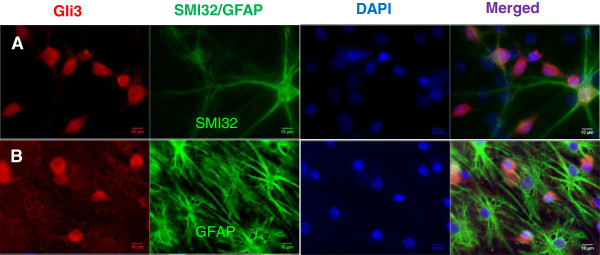
**The effect of Shh on Gli3 expression of motor neurons in culture.** After treated with Shh, every single SMI32+ cells co-stained with Gli3 **(A)**, but none of GFAP+ cells was found to co-stain with Gli3+ **(B)**. Scale bar = 10 μm **(A, B)**.

In canonical Shh signaling, Shh action is transduced by the downstream DNA-binding Gli proteins. To assess the efficacy of experimental modulation of Shh signaling in these mixed embryonic spinal cord cultures from WT and mSOD mice, we added Shh or cyclopamine to the culture media and stained for Gli3 at d2 till d11. As expected, for both WT and mSOD cultures, Gli3 staining was increased in Shh treated cultures and reduced in cyclopamine-treated cultures. When co-stained with SMI32 or GFAP (a marker of astrocytes), all SMI32 labeled cells stained for Gli3 (Figure 1A), suggesting that the Shh pathway is active in mature motor neurons expressing phosphorylated neurofilaments. In contradistinction, no GFAP-positive cells in culture expressed Gli3 (Figure 1B). We conclude that Shh is bioactive in primary motor neurons in culture and this activity may be modulated by addition of Shh or cyclopamine.

### Shh has trophic effects on motor neurons

**Figure 2:**
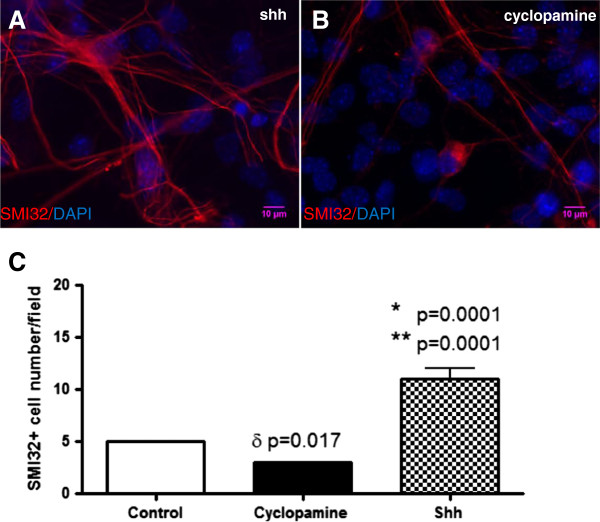
**Shh has trophic effects on motor neurons.** Representative microscopic images show SMI32+ cells from WT mice treated with Shh 250 ng/ml had larger somata and more arborization **(A)** compared to cyclopamine-treated cells **(B)**. It was shown that the average number of SMI32+ cells per field after treated with 250 ng/ml Shh was significantly higher than that control and cyclopamine (Shh 11 ± 1 vs control 5 ± 0, ** p = 0.0001; Shh 11 ± 1 vs cyclopamine 3 ± 0, * p = 0.0001; cyclopamine 3 ± 0 vs control 5 ± 0, δ p=0.017; **C)**.

We then focused on the effects of Shh or cyclopamine on motor neuron morphology and number. We treated WT and mSOD cultures with Shh (250 or 500 ng/ml) or cyclopamine (5 μM or 10 μM) between d2 and d10 and then examined SMI32 staining. At this time in culture, SMI32+ cells had long, ramified processes that stained intensely. Groups of positive cells were also present in neurospheres. Cultures treated with Shh 250 or 500 ng/ml had greatly increased SMI32 staining compared to control and especially compared to cells treated with cyclopamine. This is illustrated in Figure 2 where SMI32 staining in cells treated with Shh 250 ng/ml (Figure 2A) are compared with those treated with cyclopamine 5 μM (Figure 2B). Shh-treated cells had larger somata and more arborization, while in cyclopamine-treated but not Shh-treated cultures, dying cells were seen. TUNEL staining confirmed these cells were dying from apoptosis (not shown).

**Figure 3:**
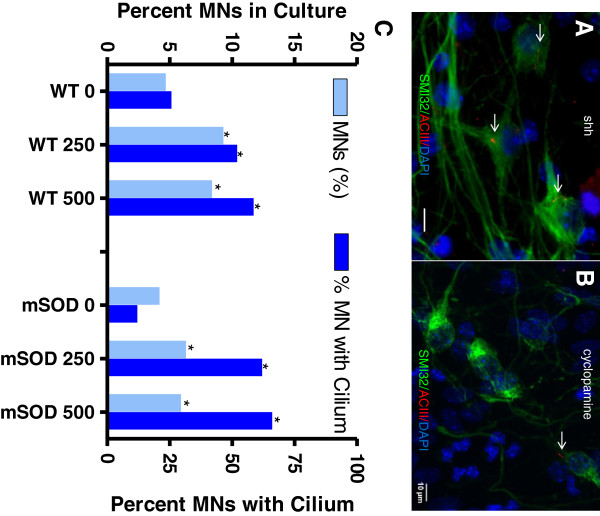
**The effect of Shh on motor neurons and motor neuron ciliation.** Representative microscopic images show there were more SMI32+ cells (green) co-stained with a cilial marker ACIII (red) in Shh 250 ng/ml treated cell culture **(B)** than in control culture **(A)** from mSOD mice. In WT cells, both doses of Shh significantly increased the percentage of motor neurons in culture and significantly increased the percentage of ciliated motor neurons (C- note the different y axes). A similar situation occurred in the mSOD cells **(C)**. * p<0.001, Shh (250 ng/ml and 500 ng/ml) vs control (0) for motor neuron percentage and ciliated motor neuron percentage in both WT and G93A SOD1 mice.

To quantity the effect of the Shh pathway on motor neuron survival, cells from WT and mSOD mice were treated between d2 and d10 with control medium or media containing Shh 250 ng/ml or cyclopamine 12.5 μM. We counted the total number of SMI32+ cells in 7–8 random low power (X 20) fields, and the results are reported as both the average number SMI32+ cells per low power field, and as the percentage SMI32+ cells relative to total cells present (DAPI). Cultures in control media averaged 5 ± 0 SMI32+ cells per low power field, while Shh had 11 ± 1 and cyclopamine 3 ± 0. The differences in these counts are highly significant as shown in Figure 2C. When compared as a percentage of total cells, similar results were seen (Figure 3C). Thus, repeated administration of Shh to mixed neuronal cultures increases the percentage and absolute number of motor neurons.

### The effect of Shh on motor neurons and motor neuron ciliation

Canonical Shh signaling occurs through the primary cilium. We have previously shown that primary cilia are present in cultured motor neurons and they are reduced on motor neurons in the spinal cord of mSOD mice [14]. We investigated whether repeated doses of Shh (250, 500 ng/ml) added to the culture media from d2 to d10 could enhance the percentage of ciliated motor neurons. At d11 cells were stained for SMI32 and the cilial marker ACIII (Figure 3A, 3B).

In WT cells, both doses of Shh significantly increased the percentage of motor neurons in culture, consistent with the above results, and significantly increased the percentage of ciliated motor neurons (Figure 3C- note the different y axes). A similar situation occurred in the mSOD cells, though if anything the effect on ciliation was more pronounced. We conclude that Shh treatment at either dose (both were roughly equivalent) increased the number of ciliated motor neurons in culture, and especially so for mSOD cells.

### The mitogenic effect of Shh on cell proliferation

Shh might increase the number of motor neurons by reducing motor neuron death or by increasing neuronal progenitor proliferation and differentiation into motor neurons. The previous experiments are too short to examine stem cell differentiation into mature (SMI32+) motor neurons (which might take 30–40 days), and the increase seen is due to reduced motor neuron death. However, analysis of BrdU staining at short and longer time frames would allow examination of the additional processes of neuronal progenitor proliferation and differentiation down motor neuron pathways.

**Figure 4:**
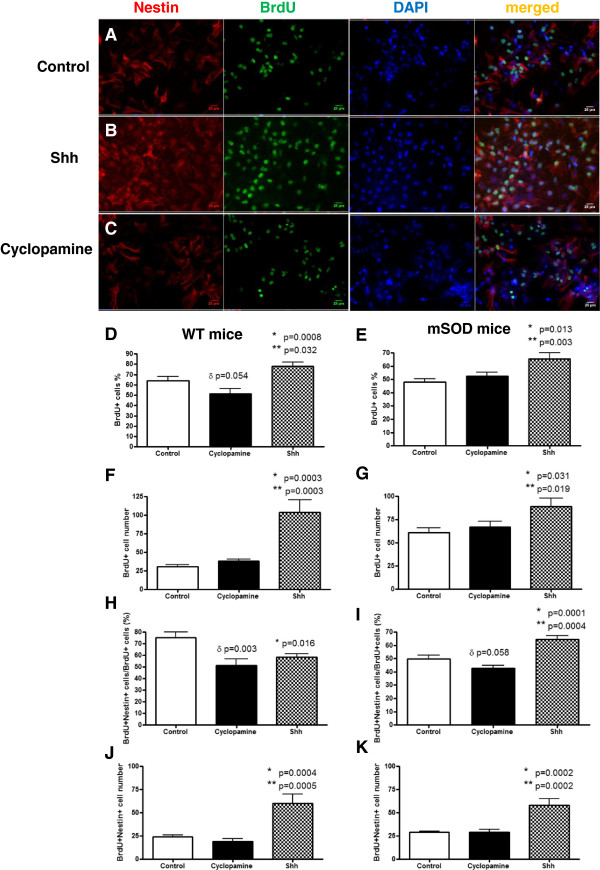
**The mitogenic effect of Shh on cell proliferation.** Representative microscopic images show BrdU labeled proliferating cells (green), nestin labeled neuronal cells (red), and doubling labeled proliferating neuronal cells of BrdU and nestin in the control **(A)**, 250 ng/ml shh treated **(B)**, and 5 μM cyclopamine treated **(C)** cell cultures from mSOD mice. With overall cell proliferation **(D, E, F, G)**, shh treated cell culture from both WT **(C, E)** and mSOD mice **(D, F)** had a significantly higher percentage **(D, E)** or number of BrdU+ cells **(F, G)** than control or cyclopamine treated cultures. With neuronal cell proliferation **(H, I, J, K)**, shh treated cell culture from both WT **(H, J)** and mSOD mice **(I, K)** had a significantly higher percentage **(H, I)** or number of double labeling of BrdU+/nestin+ cells **(J, K)** than control or cyclopamine treated cultures. The effect of cyclopamine was absent or relatively subdued in G93A SOD1 mice. ** shh vs control, * shh vs cyclopamine, δ cyclopamine vs control Scale bar =25 μm **(A, B, C)**.

We first examined the effect of Shh modulation on cell proliferation by examining BrdU incorporation into cells in culture 24 hours after a single dose of BrdU (Figure 4). There was a robust effect of Shh in both WT and mSOD cultures (Figure 4A, 4B, 4C). The effect of cyclopamine was absent or relatively subdued. When quantified, Shh treated WT (Figure 4D, 4F) and mSOD (Figure 4E, 4G) cultures had a significantly higher percentage or number of BrdU+ cells than control or cyclopamine treated cultures. We conclude that Shh treatment increases cell proliferation in both WT and mSOD cultures.

We next examined the effect of Shh modulation on neuronal progenitor proliferation by examining BrdU+ cells staining for Nestin, an intermediate filament protein and neuronal progenitor marker, 24 hours after a single dose of BrdU (Figure 4). The effects of Shh but not cyclopamine in both WT and mSOD cultures were obvious (Figure 4A, 4B, 4C). When quantified, Shh treated WT (Figure 4H, 4J) and mSOD (Figure 4I, 4K) cultures had a significantly higher percentage and number of double-labeled BrdU+/nestin+ cells than cyclopamine treated cultures or control cultures (Figure 4H, 4I, 4J, 4K). We conclude that Shh treatment increases proliferation of neuronal progenitor cells in both WT and mSOD cultures.

### The effect of Shh on cell survival and differentiation

**Figure 5:**
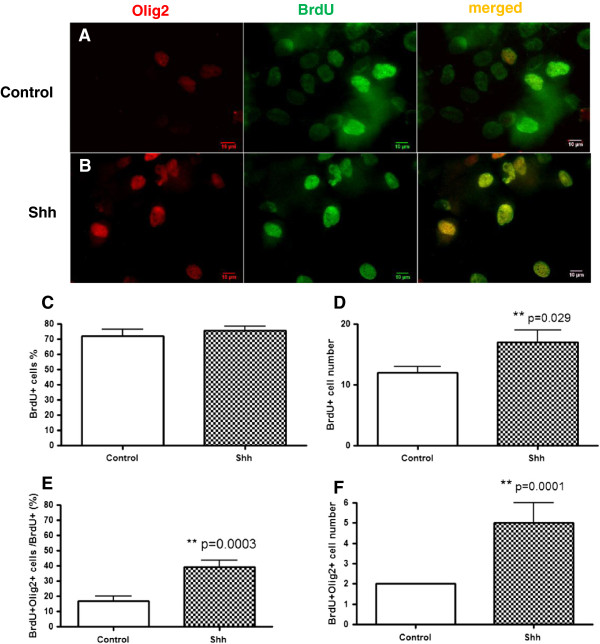
**The effect of Shh on cell survival and differentiation.** Representative microscopic images show BrdU labeled surviving cells (green), Olig2 labeled motor neuron precursor cells (red), and doubling labeled BrdU and Olig2 cells in control **(A)** and in 500 ng/ml shh treated cell culture **(B)** from WT mice. The number **(D)** but not percentage **(C)** of BrdU labeled surviving cells was significantly higher in Shh treated cultures than in controls. Both the percentage and the number of cells double-labeled with Olig2 and BrdU were significantly higher in Shh treated cultures than in controls (Shh 39.2% ± 4.6% vs control 17.0% ± 3.0%, ** p = 0.0003; Shh 5 ± 1 vs control 2 ± 0, ** p = 0.0001, **E**, **F**). Scale bar =10 μm **(A, B)**.

Neuronal progenitors differentiate and mature into motor neurons, during which they express a progression of markers [16]. Commonly employed markers include (in order) Islet1, Olig2, Hb9, and SMI32. We first examined the effect of Shh on cell survival in WT culture by counting BrdU labeled cells 2 weeks after BrdU was removed (Figure 5A, 5B). Shh administration led to a significant increase in the number of BrdU+ cells (Figure 5D). The effect was seen mainly for cell counts rather than percentage of total cells, Figure 5C.

We stained for Islet1 and Olig2, but we formally counted only Olig2, further downstream from Islet1. As expected, far fewer (BrdU+/Olig2+) double-labeled cells were detected (Figure 5A, 5B). Both the percentage and the number of cells double-labeled with Olig2 and BrdU was significantly higher in Shh treated cultures than in controls (Shh 39.2% ± 4.6% vs control 17.0% ± 3.0%, p = 0.0003; Shh 5 ± 1 vs control 2 ± 0, p = 0.0001, Figure 5E, 5F).

We also examined Hb9, but at the time point we chose, there were very few BrdU+/Hb9+ double-labeled cells, such that quantification was not feasible. Furthermore, we saw no BrdU+ cells staining for SMI32. This is expected since previous studies [16,17] estimate that it takes 12 days for neural stem cells to differentiate into Olig2+ cells, but 4–5 weeks for them to express Hb9. We conclude that Shh treatment leads to neuronal progenitor proliferation and differentiation down motor neuron lineages, but insufficient time elapsed for us to see mature motor neurons develop. (It is difficult to maintain robust motor neurons in mixed spinal cord in culture for more than a few weeks without antibiotics to prevent bacterial overgrowth, and motor neurons from mSOD cultures are particularly susceptible to antibiotic-induced damage).

## Discussion

We hypothesized that Shh might be trophic to motor neurons in primary embryonic spinal cord culture, and over a longer time frame, facilitate the proliferation and differentiation of stem cells along motor neuronal lineages. Evidence supporting both possibilities was seen.

In the short term, Shh reamins biologically active in terminally-differentiated motor neurons and reduces motor neuron death relative to other cells, while cyclopamine increases it. This trophic effect is seen in both WT and mSOD cultures.

Canonical Shh signaling requires the integrity of the primary cilium, and the percentage of ciliated motor neurons increases more so than the percentage of motor neurons in total, especially in mSOD cultures. This could be explained if Shh had a trophic effect on cilia, or perhaps more likely, if there were a preferential retention of ciliated motor neurons, in which Shh signaling is more efficient and the trophic effect of Shh greater.

The reduction in motor neuron death suggests that Shh augmentation could play a therapeutic role in ALS, where the dysfunction and death of motor neurons are central. However, the extrapolation of cell death in these embryonic cultures to the cell death characterizing late onset neurodegenerative disease must be made with caution. There is considerable variability in in-vitro systems and it remains unclear that the processes of cell death in culture faithfully mirror neurodegenerative cell death. Nonetheless, it is now clear that Shh signaling remains active in adult neurons. Shh has been shown to regulate pre-synaptic terminal size and modulate pre-synaptic function in cultured hippocampal neurons [18]. Our results are consistent with previous results demonstrating a trophic effect of Shh in models of Parkinson’s Disease [8,10], stroke [19-21], diabetic neuropathy [12,22], and spinal injury [23-26]. They are consistent with our previous studies in ALS culture models using N2A cells exposed to oxidative challenge [13]. As such, the Shh signaling pathway retains interest as a therapeutic target in neurodegenerative disease.

Shh increases stem cell proliferation in short term culture and markers of differentiation along motor neuronal lines in longer term culture. The increase in BrdU/Olig2 double-stained cells may be due to an increase in production of Olig2+ cells, a reduction in Olig2+ cell death, or both. Future studies quantifying cell death might be able to distinguish between these possibilities. The failure to see increased markers of mature motor neurons in surviving cells likely reflects the time needed for full differentiation of motor neurons, which might take 5 weeks or more in vitro [17]. However, we have found it difficult to extend the mSOD cultures more than a few weeks. This may relate to factors related to expression of the mutant transgene, to an increased sensitivity of these cells to antibiotics, or other factors. Since there is no adequate way of distinguishing these possibilities, we cultured without antibiotics, and longer term studies were undertaken only in WT cells.

It has been a long hope that stem cell therapy might be beneficial in neurodegenerative disease in general and ALS in particular. Most effort has been placed on delivery of exogenous stem cells. However, the support and targeted differentiation of endogenous stem cells might also be beneficial. In spinal cord injury and stoke, Shh treatment by a variety of delivery approaches [23-26] improves functional recovery, an effect mediated, at least in part, by expansion of oligodendroglial/motor neuronal precursors [24]. Our results suggest that the Shh-induced differentiation and maintenance of motor neuronal precusors occurs in ALS models and may be a fruitful approach in ALS.

## Conclusion

Shh provides trophic support for spinal motor neurons in primary culture derived from WT and G93A SOD1 mice. Shh also increases production of motor neuronal progenitor cells in these culture systems. These attributes could prove useful in therapeutic approaches to adult motor neuron diseases, and suggest that the Shh pathway remains an interesting therapeutic target in ALS. Further studies of Shh in ALS models are warranted.

## Abbreviations

ACIII: Adenylyl cyclase type 3; ALS: Amyotrophic lateral sclerosis; BDNF: Brain derived neurotrophic factor; BrdU: Bromodeoxyuridine; DMSO: Dimethyl sulfoxide; CNTF: Ciliary neurotrophic factor; GFAP: Glial fibrillary acidic protein; Hb9: Homeobox gene Hb9; mSOD- G93A: mutant human SOD1; N2A cells: Neuro 2A cells; Olig2: Oligodendrocyte transcription factor 2; Shh: Sonic hedgehog; SMI 32: Neurofilament H non-phosphorylated antigen; TUNEL: Terminal deoxynucleotidyl transferase dUTP nick end labeling; WT: Wildtype.

## Competing interests

The authors declare that they have no competing interests.

## Authors’ contributions

XM cultured the primary neurons, performed all the cilial counts and cell survival counting, took the photomicrographs of cultured cells, and performed the analysis of these data. She contributed to writing the paper. PT performed cell counting for cell proliferation and did genotyping. RP set up embryonic spinal cell culture and did genotyping for cultures. JT conceived the experiments, wrote, and edited the paper. All authors read and approved the final manuscript.
